# Damage-Associated Molecular Patterns in Myocardial Infarction and Heart Transplantation: The Road to Translational Success

**DOI:** 10.3389/fimmu.2020.599511

**Published:** 2020-12-08

**Authors:** Max J. M. Silvis, Selma E. Kaffka genaamd Dengler, Clémence A. Odille, Mudit Mishra, Niels P. van der Kaaij, Pieter A. Doevendans, Joost P. G. Sluijter, Dominique P. V. de Kleijn, Saskia C. A. de Jager, Lena Bosch, Gerardus P. J. van Hout

**Affiliations:** ^1^ Department of Cardiology, University Medical Center Utrecht, Utrecht, Netherlands; ^2^ Department of Cardiothoracic Surgery, University Medical Center Utrecht, Utrecht, Netherlands; ^3^ Department of Cardiology, Laboratory of Experimental Cardiology, University Medical Center Utrecht, Utrecht, Netherlands; ^4^ Central Military Hospital, Utrecht, University Medical Center Utrecht, Utrecht, Netherlands; ^5^ Netherlands Heart Institute, Utrecht, The Netherlands; ^6^ UMC Utrecht Regenerative Medicine Center, Circulatory Health Laboratory, University Utrecht, University Medical Center Utrecht, Utrecht, Netherlands; ^7^ Department of Vascular Surgery, University Medical Centre Utrecht, Utrecht, Netherlands; ^8^ Center for Translational Immunology, University Medical Center Utrecht, Netherlands

**Keywords:** ischemia reperfusion injury, myocardial infarction, heart transplantation, damage-associated molecular patterns, pattern recognition receptors, innate immunity, sterile inflammation

## Abstract

In the setting of myocardial infarction (MI), ischemia reperfusion injury (IRI) occurs due to occlusion (ischemia) and subsequent re-establishment of blood flow (reperfusion) of a coronary artery. A similar phenomenon is observed in heart transplantation (HTx) when, after cold storage, the donor heart is connected to the recipient’s circulation. Although reperfusion is essential for the survival of cardiomyocytes, it paradoxically leads to additional myocardial damage in experimental MI and HTx models. Damage (or danger)-associated molecular patterns (DAMPs) are endogenous molecules released after cellular damage or stress such as myocardial IRI. DAMPs activate pattern recognition receptors (PRRs), and set in motion a complex signaling cascade resulting in the release of cytokines and a profound inflammatory reaction. This inflammatory response is thought to function as a double-edged sword. Although it enables removal of cell debris and promotes wound healing, DAMP mediated signalling can also exacerbate the inflammatory state in a disproportional matter, thereby leading to additional tissue damage. Upon MI, this leads to expansion of the infarcted area and deterioration of cardiac function in preclinical models. Eventually this culminates in adverse myocardial remodeling; a process that leads to increased myocardial fibrosis, gradual further loss of cardiomyocytes, left ventricular dilation and heart failure. Upon HTx, DAMPs aggravate ischemic damage, which results in more pronounced reperfusion injury that impacts cardiac function and increases the occurrence of primary graft dysfunction and graft rejection via cytokine release, cardiac edema, enhanced myocardial/endothelial damage and allograft fibrosis. Therapies targeting DAMPs or PRRs have predominantly been investigated in experimental models and are potentially cardioprotective. To date, however, none of these interventions have reached the clinical arena. In this review we summarize the current evidence of involvement of DAMPs and PRRs in the inflammatory response after MI and HTx. Furthermore, we will discuss various current therapeutic approaches targeting this complex interplay and provide possible reasons why clinical translation still fails.

## Introduction

Acute myocardial infarction (MI) is typically the result of hampered blood flow to the myocardium due to atherosclerotic plaque rupture or erosion (1). Consequential to hampered blood flow, the myocardium becomes ischemic with subsequent loss of viable cardiac muscle. Timely and adequate reperfusion leads to limitation of the infarct size (IS), partial preservation of cardiac function and subsequent reduction of morbidity and mortality (1). In heart transplantation (HTx)-ischemic time has been linked to primary graft dysfunction and rejection. To limit ischemic injury as much as possible, the donor heart is arrested with a cardioplegic solution and stored on ice in the same solution. Hypothermia reduces the metabolic demands and thereby protects the tissue from acute deprivation of nutrients. Unfortunately, this protective effect is limited by time. Timely restoration of blood flow is therefore also essential for preservation of cardiac function and thereby outcome of HTx (2–4).

Although reperfusion is the single most effective treatment in salvaging the ischemic area at risk, it also causes additional cardiomyocyte death (5). This phenomenon is known as ischemia reperfusion injury (IRI) and is characterized by initial intracellular metabolic changes that amplify damage (1, 5). During the (hyper)acute phase of reperfusion (seconds to minutes), disruption of electron transport leads to intracellular calcium overload and reactive oxygen species (ROS) formation. This induces opening of mitochondrial permeability transition pores culminating in increased mitochondrial calcium levels, subsequent mitochondrial membrane rupture, and eventual cell death.

In the following minutes to hours and even days, cell damage and cell death, provoke a burst of cellular components (e.g. Heat Shock Proteins (HSPs), High Mobility Group Box-1 (HMGB-1), Adenosine Triphosphate (ATP), nuclear and mitochondrial DNA (mtDNA), and RNA) into the extracellular space and the circulation where these molecules act as so called damage (or danger)-associated molecular patterns (DAMPs). The constitutive molecules that are excessively released during cell injury or cell death have also been called alarmins but the terms DAMPs and alarmins have been used interchangeably in various studies (6). DAMPs are essential activators of the complex signaling cascade that eventually leads to reperfusion induced cardiac damage. DAMPs serve as ligands for pattern recognition receptors (PRRs) that, when activated, induce nuclear translocation of various transcription factors (e.g. Nuclear Factor kappa-light-chain-enhancer of activated B cells (NF-κB)) and promote pro-inflammatory cytokine release (7, 8).

**Figure 1:**
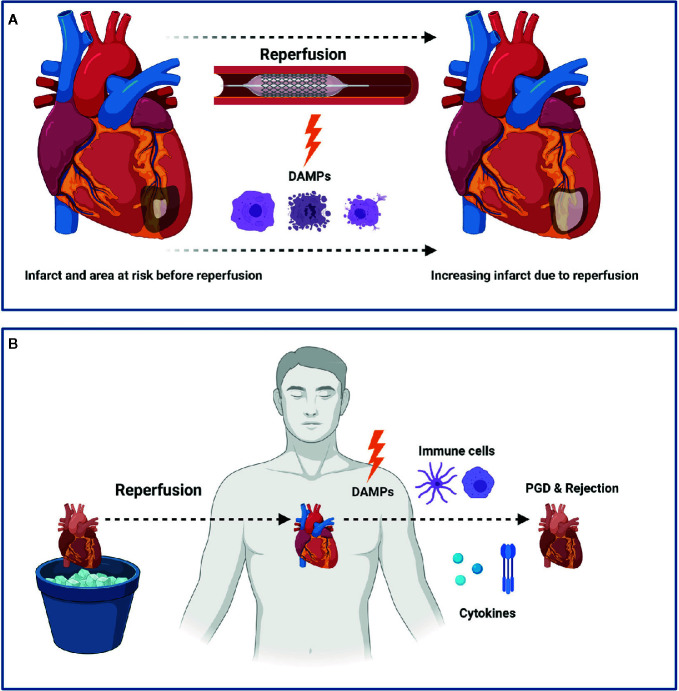
Myocardial Ischemia Reperfusion Injury (IRI) following myocardial infarction (MI) or heart transplantation (HTx). IRI leads to the release of damage associated molecular patterns (DAMPs) which subsequently leads to an increased infarct size following MI **(A)**. In HTx, the release of DAMPs leads to a pro-inflammatory state that ultimately can lead to primary graft dysfunction (PGD) and rejection **(B)**. Figure was created with BioRender.com.

Targeting the inflammatory response after myocardial IRI has in part proven to be successful in small and large animal models, whereas clinical translation has proven to be very challenging and cumbersome (9). In this review we summarize the current evidence of involvement of PRRs and DAMPs in the inflammatory response induced by IRI in the light of MI and HTx (summarized in **Figure 1**). Furthermore, we will discuss the various current therapeutic approaches targeting this complex interplay and provide possible reasons why these therapies are currently not successfully translated to clinical therapies.

## Damage-Associated Molecular Patterns Related to Myocardial Ischemia Reperfusion Injury

Multiple DAMPs play a role in myocardial IRI (summarized in **Table 1**) and they are extensively studied in experimental MI models (70, 71). The goal of these models is to mimic clinical IRI as closely as possible and investigate possible DAMP-related therapies as closely as possible. The primary outcome measurements are typically myocardial IS and cardiac function following ischemia and reperfusion (5). DAMPs and IRI are investigated less intensively in HTx (72). However, targeting DAMPs, was studied in kidney, liver and lung transplantation, aiming for a reduction of primary graft dysfunction and rejection (73–76). Here we will discuss the most relevant DAMPs and PRRs and therapies targeting their interplay in the setting of myocardial IRI following both MI and HTx (summarized in **Table 2**).

**Table 1:** DAMPs and PRRs related to MI and HTx.

DAMP	Ligand for	DAMP’s effect in MI		DAMP’s effect in HTx	
		Protective	Detrimental	Protective	Detrimental
HMGB-1	RAGE, TLR2, TLR4, TLR9 (10–14)	↑ cardiac output, ↓IS (15–18)	↓ cardiac output (18) ↑ IRI (11)	–	↑ inflammation, ↑ rejection (19–21)
HSP60	TLR2, TLR4 (22, 23)	–	↑ inflammation, ↑ damage (24–26)	↓rejection (27)	–
HSP70	TLR2, TLR4	↓IS (28)	↑ inflammation, ↑ IS (29) ↑ adverse remodeling, ↑ IS (30)	↑ contractility, ↑ endothelial and mitochondrial function (31, 32) ↓ rejection (33)	–
Fibronectin-EDA	TLR2, TLR4 (34–36)	–	↑ adverse remodeling (35) ↑ heart failure (35) ↑ IRI (36)	–	↑ rejection (37, 38) ↑ inflammation (39)
Hyaluronic acid	TLR2, TLR4 (40, 41)	↑ cardiac function, ↓IS (42, 43)	↑ edema (44) ↑ damage (45)	–	↑ edema, ↑ rejection (46) ↑ inflammation, ↑ fibrosis (47)
mtDNA	TLR9, NLRP3, RAGE (12, 48–50)	–	↑ mtDNA replication, ↑ damage (51, 52) ↑ inflammation (12, 50, 53) ↑ IS (53)	–	–
Circulating RNA	TLR3, TLR7 (54, 55)	–	↑ inflammation (55–57) ↑ IS (56–58)	–	–
Cardiac myosin	TLR2, TLR8 (59)	–	↑ inflammation (59)	–	↑ rejection (60, 61) ↓ graft survival (62, 63)
Extracellular ATP	P2X_7_/NLRP3 (64–66)	–	↑ IS (67) ↓ cardiac function (68)	–	–
Calgranulins	RAGE (14, 69)	–	↓ recovery (69)	–	–

**Table 2:** Experimental therapeutics.

Target	Compound	Compound effect on primary outcome			
		MI	HTx	Other transplant	Human studies
HMGB-1	Anti-HMGB-1 antibody	↑ IS (16) ↑ infarct scar (17)	↑ graft survival, ↓inflammation (19–21)	–	–
	BOX A	↓IS (11)	–	–	–
	Glycyrrhizin	↓IRI/IS (77, 78) ↓ inflammation (77, 78)	↑ graft survival, ↓inflammation (79, 80)	–	–
HSP70	Bimoclomol	↓ IS (28)	–	–	–
	GGA	↓IRI (81, 82)	–	–	↑HSP concentration (83)
Fibronectin-EDA	Anti-Fibronectin-EDA antibody	↓ IS (36)	–	–	–
mtDNA	Epigallocatechin-3-gallate (EGCG)	↓ IS, ↓ inflammation (53)	–	–	–
	Exscien-1-III	↓ remodeling, ↑ cardiac function (84)	–	–	–
exRNA	RNase	↓ IS, ↓ inflammation, ↓necrosis (56, 58)	–	–	–
	Dex-TO	↓ IS, inflammation (57)	–	–	–
TLR4	Eritoran	↓ IS and inflammation (85)	–	↓ inflammation (86)	= inflammation (87)
	TAK-242	↓ IS (88)	–	–	= inflammation (89)
TLR2	OPN-305	↓ IS, ↓ inflammation, ↓remodeling, ↑ cardiac function (90, 91)	–	↑ Renal function (92)	↓ IL-6, no adverse effects (93)
NLRP3	INF4E	↓ IS, ↑ cardiac function (94)	–	–	–
	16673-34-0	↓ IS and inflammation (95)	↑ graft function (96)	–	–
	Dapansutrile	↓ IS, ↓inflammation, ↑ cardiac function (97)	–	–	↓ IL-1β, Safe, well-tolerated (98–100)
	MCC950	↓ IS, ↑ cardiac function (101)	–	–	–
P2X_7_	shRNA	↓ IS, ↓ inflammation, ↑ cardiac function (67)	–	–	–
	siRNA (NONRATT021972)	↑ cardiac function (102)	–	–	–
	PPADS	↓ IS, ↓ cell death, ↑ cardiac function (68)	–	–	–
	Genestin	↓ IS, ↓inflammation (103)	–	–	–
	Periodate-oxidized ATP	–	↓ rejection (104)	–	–
RAGE	sRAGE/A + genetic silencing of RAGE	↓ IS and fibrosis (105)	–	–	–
NF-κB	BAY 11-7082	↓ IS, ↓inflammation (106)	–	–	–
	BAY 65-1942	↓ IS, ↓inflammation ↑ cardiac function (107)	–	–	–
	PS-519	↓ IS, ↓inflammation ↑ cardiac function (108)	–	–	–
	Tacrolimus	↓ myocardial necrosis ↓inflammation (109)	–	–	–
	PDTC	–	↑ graft survival (110)	↓IRI lung transplant (111)	–

### High Mobility Group Box 1

High mobility group box 1 (HMGB-1) is a nuclear protein that regulates transcription, DNA replication and DNA repair. Consequently, cells lacking HMGB-1 have an increased susceptibility to DNA damage. Upon cell stress or injury, HMGB-1 can be passively or actively released (112). Outside the cell, HMGB-1 induces an immune response by acting as a DAMP on multiple PRRs (113). The first receptor found to interact with HMGB-1 was the receptor for advanced glycation end-products (RAGE), but HMGB-1 can also act as a DAMP on both Toll-like receptor (TLR)2 and TLR4 in neutrophils and macrophages (10).

Following these observations, rodent studies focused on the role of HMGB-1, in MI and HTx. Surprisingly, overexpression of HMGB-1 in MI models of permanent ligation and administration of HMGB-1 after infarct induction resulted in the attenuation of dendritic cell influx, smaller IS and the preservation of left ventricular ejection fraction (15, 16). These beneficial effects were further accompanied by enhanced angiogenesis (16). Similarly, administration of anti-HMGB-1 in rats, both in a MI model of permanent ligation and an IRI model led to an enlarged IS and thinning/expansion of the infarct scar, suggesting a possible essential role for HMGB-1 in the healing process after MI (17, 18).

Conversely, the opposite was observed in a study by Andrassy et al., where recombinant HMGB-1 enhanced cardiac TNF-α and IL-6 mRNA levels, increased IS and worsened IRI. In line with these findings, HMGB-1 box A, a functional antagonist of extracellular HMGB-1 and HMGB-1 interaction with RAGE, led to a pronounced reduction of IS (11). HMGB-1 was also studied in humans and showed to be significantly elevated in serum after acute MI, within 24h of symptom onset, compared to healthy volunteers (114). Furthermore, higher peak levels were associated with a higher incidence of adverse cardiac outcomes, such as pump failure, cardiac rupture, and in-hospital cardiac death (18).

In HTx models, the use of anti-HMGB-1 neutralizing antibody during whole-heart cold reperfusion reduced systemic release of pro-inflammatory cytokines in the mouse allograft (19). In a mouse model of acute rejection, blockade of HMGB-1 significantly decreased infiltration of neutrophils and alloreactive T helper 17 (Th17) cells leading to prolonged allograft survival (20). Furthermore, the administration of a specific anti-HMGB-1 monoclonal antibody in a mouse model of chronic rejection decreased cardiac allograft vasculopathy and fibrosis. This was accompanied by attenuation of T-cell infiltration, reduced numbers of inflammatory dendritic cells together with decreased Th17 and interferon-gamma production in the allograft and the recipient’s spleen (21). These results suggest that anti-HMGB-1 treatment may improve both short and long term HTx outcome.

Glycyrrhizin (GL) is a natural inhibitor of extracellular HMGB-1 and inhibits the formation of pro-inflammatory cytokines (77, 78). In a rat model of MI, the use of GL dose- dependently attenuated IRI (78). When added to the preservation solution during cold storage in a HTx mouse model, GL improved cardiac allograft function and resulted in less myocardial damage (79). Additionally, intraperitoneal administration of GL to the mouse recipient, 5 min before reperfusion in a HTx mouse model, markedly reduced the production of interleukin (IL)-23 and IL-17A and decreased cardiac troponin T and cardiomyocyte apoptosis (80).

It has been postulated that discrepant results in different animal models of MI (permanent ligation vs. IRI) indicate that HMGB-1 elicits both beneficial (promoting-angiogenesis in chronic repair) and harmful effects (IRI) following MI. These heterogeneous results make it challenging to determine the therapeutic window for beneficial effects and hinders clinical translation in the setting of MI. Therefore, an improved understanding of the release mechanism of HMGB-1 as well as the downstream effects in the light of IRI following MI or HTx will be critical for elucidating the various physiological roles of HMGB-1 in cardiovascular disease. In summary, given the complexity of the role of HMGB-1, it is unlikely that selective HMGB-1 (ant)agonists will reach the clinical area.

### Heat Shock Proteins

Heat shock proteins (HSPs) are chaperone proteins that organize the folding, assembly, and degradation of cellular proteins and are present in all cells. HSPs were initially defined as proteins released in response to heat shock and they are further grouped into families based on their molecular weight (115, 116). Particularly HSP60 and HSP70 are associated with IRI in MI and HTx. It is debatably whether HSPs are true DAMPs, since detectable levels of certain HSPs exist in unprovoked extracellular environments, while DAMPs are intracellular molecules, expressed upon stress or damage, that normally are not exposed to the extracellular environment (117).

Exposure of rodent cardiomyocytes to exogenous HSP60 induces apoptosis and leads to a significant increase of inflammatory cytokines through TLR4 and TLR2 mediated signaling (22, 23). Transient myocardial ischemia results in a pronounced release of HSP60 and is associated with cardiac inflammation and damage *in vitro* and *in vivo* (24–26). Furthermore, the expression of HSP60 following MI seems to differ over time suggesting different significance in the acute phase following IRI and (sub)acute phase of adverse remodeling and progression to heart failure (118).

The role of HSP70 in MI has shown to be complex since opposed results have been published. HSP70 can induce the release of pro-inflammatory cytokines in human monocytes (29). However, pharmacological treatment with HSP co-inducer Bimoclomol increased levels of HSP70, but decreased IS in a rat MI model of IRI (28). Additionally, induction of HSP70 expression following pretreatment with pharmacological agent Geranylgeranylacetone (GGA), also revealed cardioprotective effects in rat models of IRI (81, 82). Moreover, a small cohort of 26 patients undergoing coronary artery bypass grafting was randomized to 3-day pre-treatment with GGA (400mg/day) or placebo. Treatment with GGA resulted in higher levels of HSP70 and other small HSPs in the myofilament fractions of right and left atrial appendage tissue. This could indicate that GGA treatment results in the upregulation of beneficial HSPs that could potentially attenuate IRI following MI (83). Importantly, both bimoclomol and GGA are indirect regulators of HSP70 and these studies fail to establish a clear causal role between upregulation of HSP70 and the observed treatment effects.

A recent study in ST-elevated Myocardial Infarction (STEMI) patients observed that serum levels of HSP70 were significantly increased following MI and were associated with increased IS, adverse remodeling and worse clinical outcome. This study indicates that HSP70 can serve as a biomarker of clinical outcome, albeit it does not prove a causal role of HSP70 as a key player of the post-MI inflammatory response (30).

Although less studied in HTx, a clinical study demonstrated that endomyocardial HSP60 levels were low just before and during acute rejection. The levels of HSP60 increased after treatment with immunosuppressive agents, suggesting that HSP60, in contrast to what was reported in MI, may be released as part of a protective response following acute tissue damage in HTx (27). Cardioprotective effects of HSP70 in HTx were reported as well. In a clinical heart preservation protocol in rats, it was observed that HSP70 gene transfection protected endothelial as well as mitochondrial function, resulting in improved ventricular contractility (31, 32). A clinical HTx study could not demonstrate HSP70 expression in cardiomyocytes of patients with rejection, whereas patients without rejection did show levels of HSP70. This suggests a possible inverse relationship between HSP70 expression and rejection, implicating cellular HSP70 could possibly serve as a marker to predict graft function (33). Similar results were also observed in clinical liver transplantation studies, where lower levels of HSP70 in biopsies prior to transplantation and organ perfusates were associated with early graft loss (119).

Taken together, contradicting evidence of HSPs exists. In the aforementioned studies no evidence is provided that the pharmacological stimulation of HSP70 release is selective. One could speculate that the beneficial results after compound administration are due to unexplored pleiotropic effects. Additionally, no clear direct molecular interaction of HSPs with specific PRRs other than TLR2 and TLR4 has been reported so far and interaction with unidentified PRRs could possibly explain the ambiguous role of these HSPs. It is not fully understood whether these HSPs are innocent bystanders that increase upon myocardial injury or are causally involved in the inflammatory response after MI. Taken together, these discrepant results uncover a gap of knowledge on the role of HSPs in both MI and HTx. Consequently, targeting HSPs directly at this stage is only feasible when mechanistic studies uncover the complex role of different HSPs in IRI.

### Fibronectin-EDA

Fibronectin is a glycoprotein that is present in the extracellular matrix. It may contain a spliced exon encoding type III repeat extra domain A (EDA). Fibronectin-EDA is believed to be predominantly released by fibroblasts, in response to injury and showed its ability to act as a DAMP via TLR2 and TLR4 stimulation (34, 35).

Deletion of fibronectin-EDA is associated with less adverse remodeling and heart failure in a MI mouse model of permanent coronary ligation (35). Fibronectin-EDA also showed to contribute to myocardial IRI via TLR4-mediated signaling in hyperlipidemic mice (36) and administration of an anti-fibronectin antibody, 15 min post-reperfusion, reduced IS following ischemia/reperfusion in the same mouse model as well (36). In HTx, rodent studies revealed involvement in chronic cardiac allograft rejection (37, 38). Fibronectin-EDA expression showed to be associated with cardiac allograft vasculopathy and fibrosis. Acute rejection, however, was similar in fibronectin-EDA-deficient compared to wildtype mice (38). Additionally, a clinical study observed an association between levels of fibronectin-EDA and inflammation in biopsies of HTx patients with signs of chronic rejection (39). The exact role of fibronectin-EDA in the pro-inflammatory response and as a therapeutic target following MI and HTx, however, needs further investigation.

### Hyaluronic Acid

Hyaluronic acid (HA, also known as Hyaluronan) is a polysaccharide that together with collagen is one of the most abundant components of the extracellular matrix and is expressed in several tissues. In humans, HA is actively produced upon tissue injury and is involved in tissue repair (120, 121). The cell signaling functions of HA largely depend on its molecular weight. In case of tissue injury, such as following MI or HTx, HA is more polydisperse and fragmented (122).

Both low and high molecular weight HA fragments can act as ligands for TLR2 and TLR4 and therefore have the potential to act as DAMPs during the inflammatory response in IRI (40, 41, 70). However, low molecular weight (LMW)-HA is known to be pro-inflammatory whereas high molecular weight (HMW)-HA has anti-apoptotic and anti-inflammatory effects (123–126).

Increased expression of HA was observed in a MI rat model of permanent ligation. HA expression showed to increase rapidly in the infarcted myocardium and was associated with increased and harmful cardiac edema (44). More recently, the role of HA in a mouse model of IRI was studied. Inhibition of HA secretion, by genetic deletion of the HA Synthase 2 (*Has2)* gene, worsened cardiac function and increased IS. This shows that the early HA response appears to be part of essential repair mechanisms after IRI, albeit this study did not elaborate on the type of HA that is responsible for these cardioprotective effects (42). Furthermore, it was shown that intravenous injections of degradative fragments of HMW-HA improve myocardial function, reduce IS and promote angiogenesis in a MI mouse model of permanent ligation. These HA oligosaccharides potentially cause this protective effect by suppressing neutrophil activity and stimulating polarization of M2 type macrophages (43). The results in these different models indicate that HA has an essential role in cardiac repair following MI and IRI and, although it can act as a ligand for TLR2 and TLR4, questions its role as a detrimental DAMP. A recently published clinical study showed that the plasma HA levels are significantly increased in patients with acute MI and indicate that it has the potential to serve as a biomarker of myocardial damage but failed to establish a causal role between HA upregulation and cardiac damage (45).

The role of HA in HTx is largely unknown and only few dated experimental studies report on the expression of HA following HTx. In a rat model of HTx interstitial accumulation of HA was associated with increased edema and cardiac allograft rejection (46). More recently, in another rat model of HTx, IRI resulted in increased expression of HA and HA synthases shortly after transplant. This increased HA was followed by a pronounced infiltration of T-cells and graft fibrosis (47).

The complex role of HA in the inflammatory response following MI and HTx, with multiple forms being both of positive and negative influence on cardiac damage following IRI, makes it a difficult direct target for future clinical translation.

### mtDNA

Mitochondrial DNA (mtDNA) can be released in the extracellular space and in the circulatory system as a consequence of cell death. It can act as a DAMP and has the ability to activate the innate immune system (127–129). MtDNA is circular and carries unmethylated deoxycytidylate-phosphate-deoxyguanylate (CpG) regions similar to bacterial DNA and is therefore easily recognized as a DAMP (127, 128). During myocardial IRI, ROS production is aggravated which leads to the oxidation of mtDNA and this subsequently induces cellular damage and secondary upregulation of genes involved in mtDNA replication, activating a positive feedback loop (51, 52). MtDNA can be recognized by TLR 9, RAGE and the NOD-, LRR- and pyrin domain-containing protein 3 (NLRP3) inflammasome and activates various pro-inflammatory signaling cascades (12, 48–50).

Pharmacological inhibition of mtDNA was studied with Epigallocatechin-3-gallate (EGCG), which is a catechin with well-known anti-inflammatory properties, in a rat model of IRI. Significant positive correlations were observed between levels of mtDNA and pro-inflammatory cytokines (TNF-α and IL-6) in the myocardial tissue of non-treated rats. Administration of EGCG, prior to reperfusion, significantly reduced the levels of mtDNA, TNF-α and IL-6 and limited IS. This suggests that the cardioprotective effects were achieved by inhibiting mtDNA release, but the mode of action is not completely elucidated (53). Another study shows that administering Exscien1-III, a mitochondria-targeted fusion protein containing endonuclease III, during reperfusion in a mouse model of MI attenuates cardiac remodeling and preserves cardiac function (84). More recently, a study in mice provided further mechanistic insight in mtDNA-induced myocardial IRI and suggests that the detrimental effects are based on a combined effect with HMGB-1. It was observed that, upon IRI, both HMGB-1 and mtDNA are released into the bloodstream. Solo-treatment of either HMGB-1 or mtDNA infusion did not result in increasing IS, while combined treatment of HMGB-1 and mtDNA did have clear harmful effects via RAGE signaling (12).

Although these results are interesting, to the best of our knowledge no clinical trials with drugs targeting mtDNA have been reported and whether mtDNA could also serve as a potential therapeutic target in patients, remains to be elucidated.

### Circulating Extracellular RNA

RNA molecules can be actively or passively secreted into the extracellular milieu following IRI and were shown to act as a DAMP by inducing pro-coagulatory and pro-inflammatory responses (130). These circulating extracellular RNA (exRNA) molecules elicit a strong immune response by stimulation of TRIF-signaling, induction of interferon secretion (independent of TLR2/4‐MyD88 signaling) and upon interaction with different TLRs, such as TLR3, TLR7 and TLR8 both *in vitro* and *in vivo* in myocardial IRI (54–56, 131, 132). It was further demonstrated that treatment with RNase attenuates necrosis-induced cytokine production in cardiomyocytes and protects mice against IRI, marked by smaller IS (56). This protective effect of exRNA inhibition was confirmed by administration of a non-toxic RNase1 which resulted in reduced IS and preserved cardiac function in an experimental *in vivo* mouse model of myocardial IRI as well as in the isolated IRI Langendorff-perfused rat heart (58).The same group tested this concept in a clinical study on patients undergoing cardiac bypass surgery and demonstrated that upon remote ischemic preconditioning, by four 5-min cycles of blood pressure cuff inflation around the left arm, prior to the surgery, the levels of cardioprotective RNase1 were increased whereas the concentration of exRNA and Tumor Necrosis Factor-α (TNF-α) were decreased. This study does not report the direct effects on cardiac function neither does it prove a causality between upregulation of RNAse1 and a reduction of exRNA and TNF-α. The exact mechanism of RNase1-induced cardioprotection, therefore, still remains to be clarified (133).

Others developed a multivalent nucleic acid scavenging nanoprobe, fluorochrome thiazole orange conjugated to a dextran carrier (Dex-TO), to scavenge nucleic acids. Dex-TO was administered intravenously to mice subjected to MI, initially at the onset of IRI and again 4 h later, resulting in a reduced inflammation and a decrease in IS in the Dex-TO treated mice (57).

These data indicate that RNA-induced tissue injury can potentially be reduced with RNase, TLR inhibitors, and other RNA scavenging chemicals compounds. However, more studies in large animals and clinical data are needed to support these beneficial effects observed in pre-clinical experimental studies.

### Cardiac Myosin

Cardiac myosin (CM) is a contractile protein that is a part of the sarcomeric complex. It is unique to the myocardium and forms the most abundant protein in the heart. CM is released from necrotic myocytes to the blood following MI and induced humoral immune responses in patients post-MI. *In vitro*, CM released by damaged cardiomyocytes, demonstrated to stimulate the innate immune response. Binding of CM to TLR2 and TLR8 resulted in the release of pro-inflammatory cytokines by monocytes which could be involved in the detrimental inflammatory response *in vivo* (59). However, its role in myocardial IRI was not further investigated.

CM is also thought to be involved in HTx as it is recognized by both T-cells and B-cells during cardiac rejection (60). Mouse models of HTx have shown that sensitization with CM prior to transplant can lead to an augmented rejection of both allogenic and syngeneic cardiac grafts (60). Modulation of the CM-specific response can also influence alloreactivity and graft survival in a mouse model of HTx (62). Higher anti-myosin IgG antibody levels post HTx have shown to be associated with acute cardiac rejection in a clinical study (61). Furthermore, high levels of anti-myosin IgG pre-transplant were shown to be linked with poor survival after HTx, and increased levels post-transplant were related to increased risk of acute rejection (63).

## Pattern Recognition Receptors Related to Myocardial Ischemia Reperfusion Injury

### Toll-Like Receptors

Toll-like receptors (TLRs) are transmembrane proteins that are expressed by a variety of cells and play a pivotal role in the innate and adaptive immune system. The immune response provoked by TLR-activation differs according to the type of the stimulus (134). It was shown that almost all cell types present in the heart express TLRs (135). TLR4 and TLR2 have been studied extensively in the light of IRI during experimental MI (136) and HTx (137).

### TLR4

Most research on TLRs has been performed on mouse strains that are deficient of certain TLRs. There is strong evidence that TLR4 contributes to IRI in both MI (138–140) and HTx (137, 141). The role of TLR4 in transplantation has also been confirmed for other solid organ transplants (142–145). Given these observations, TLR4 inhibition may have beneficial effects in limiting IRI. Several compounds targeting TLR4 have been developed and some were tested in myocardial IRI upon MI or organ transplantation. In this perspective, it was shown that pretreatment with the TLR4 antagonist Eritoran reduced IS by 33% in a MI mouse model of IRI (85). Additionally, the release of TNF-α, IL-6, and IL-1β was significantly attenuated. Another TLR4-antagonist that has been studied in a MI mouse model of IRI is TAK-242. Incorporation of TAK-242 into poly-(lactic-co-glycolic acid) nanoparticles (TAK-242-NP), was shown to improve drug delivery to monocytes and macrophages compared to non-encapsulated TAK-242. Administration of these TAK-242-NP resulted in a significant reduction of IS (88). Although these drugs have not been tested in models of HTx, Eritoran showed to reduce monocyte infiltration and pro-inflammatory cytokine production (TNF-α, IL-1β, and IL6) upon renal transplantation with 40 min of ischemia in rats (86). Eritoran and TAK-242 have not been studied in large animal models for IRI in MI or HTx, but were tested in patients as a possible treatment for sepsis (87, 89). Both drugs failed to reach hard clinical end points and overall circulating cytokine levels were unaffected.

### TLR2

Comparable to TLR4, the causal role for TLR2 in MI related IRI was established in TLR2 deficient mice (146, 147). The promising findings resulted in the development and investigation of a monoclonal anti-TLR2 antibody OPN-305 (OPN-301 in mice). Antagonizing TLR2 led to a reduction in IS and preservation of cardiac function and geometry upon cardiac ischemia reperfusion in a mouse model of IRI. This effect was attributed to reduced influx of leukocytes, decreased cytokine production and enhanced cardiomyocyte survival (90). In an open chest pig model of myocardial IRI, the administration of OPN-305 resulted in a dose-dependent reduction of IS (45% reduction with the highest dose of OPN-305) and preserved systolic function. However, the authors did not observe signs of reduced myocardial inflammation (91).

In contrast with the consistent results in MI, the role of TLR2 in HTx seems to be more ambiguous. Increased messenger RNA expression of TLR2 in the cardiac allograft is associated with increased risk of cardiac allograft rejection in humans (141). This is in line with earlier studies showing similar findings in kidney transplantation (143, 144). A recent study using TLR2-deficient mice on the other hand showed poor graft survival time, due to increased immune cell infiltration and enhanced pro-inflammatory Th17 cell responses (148). OPN-301 was not tested in models of HTx but was studied in a mouse model of renal transplantation, where it was administered after 30 min of cold ischemia prior to reperfusion. This treatment significantly improved kidney function after 6 days of transplantation based on blood urea nitrogen levels, which correlated with preserved tubular structure in mice that were treated with OPN-301 (92).

The first translational steps with OPN-305 were set with a randomized, double-blind, placebo controlled, dose escalating Phase 1 trial. In this trial a single dose of increasing concentration of the antibody was administered to study pharmacokinetics and – dynamics in 41 healthy subjects. TLR2 blockade was confirmed in a dose-dependent manner and as a consequence, inhibition of IL-6 release was observed without adverse events (93). Unfortunately no data is published on the placebo-controlled clinical trial to evaluate the efficacy of OPN-305 in delayed renal graft function that was completed in 2013 (149).

### Other TLRs

Although other TLRs are investigated less extensively, some of these receptors showed to be involved in inflammation following MI. Mice deficient for TLR3 and TLR7 showed to develop smaller IS, had less influx of inflammatory cells and better cardiac function compared to wildtype mice (150, 151). Recently, TLR9 also showed to contribute to the inflammatory response following MI in a mouse model through stimulation by HMGB-1 and mtDNA (12). However, a contrary report observed that TLR9-HMGB-1 was essential for survival of cells, wound healing and angiogenesis in a model with a longer follow-up (13). Experimental studies investigation the role of TLR5 were also performed and it is expressed in the hearts of mice, rats and humans (152). TLR5 deficiency in knock out models increased IS and myocardial oxidative stress, suggesting that TLR5 is more involved in cardioprotective signaling (153). The role of TLR8 is not completely clear but human cardiac myosin, once released after cardiomyocyte damage, can act as a ligand for TLR8 (59). Whether the pathways that are induced via other TLRs contribute to, or hamper cardiac IRI is not fully substantiated and warrants further investigation before these PRRs can be evaluated as therapeutic targets.

### NOD-Like Receptors

The nucleotide-binding oligomerization domain-like receptors, or NOD-like receptors (NLRs) are intracellular receptors that can recognize a wide variety of DAMPs. Some are known to form multi-protein complexes named “inflammasomes” (154).

Activation of the inflammasome is a two-step process of priming and activation. The priming signal is provided by the interaction of DAMPs with PRRs, such as TLRs, and leads to the upregulation of the separate inflammasome components via NF-κB activation (64). The activation signal is again provided by multiple DAMPs, including exATP and intracellular DAMPs like ROS (64). This leads to the assembly of the multiple components of the inflammasome resulting in a conformational change of caspase-1. In turn, this enables maturation and release of the cytokines interleukin (IL)-1β and IL-18, thereby inducing a potent inflammatory response (64). Additionally, caspase-1 induces pyroptosis, which is a pro-inflammatory form of regulated cell death. The best studied inflammasome is the NLRP3 inflammasome and it has been associated with cardiovascular disease extensively (155–157). It consists of three components (1): NLRP3 (2), Apoptosis-associated speck like protein containing a caspase-recruitment domain (ASC), and (3) caspase-1. Knock-out models and silencing RNA administration of NLRP3-inflammasome components revealed a possible role for the NLRP3 inflammasome in myocardial IRI (68, 158). Furthermore, IRI showed to induce a strong upregulation of NLRP3 and caspase-1 expression in the mouse heart (159). These experimental findings led to the development of selective NLRP3 inhibitors that were tested in animal models of IRI. Several compounds showed to preserve cardiac function and reduce IS.

Pretreatment with INF4E, a compound that inhibits the ATPase activity of NLRP3, reduced IS and improved postischemic left ventricular pressure in isolated male Wistar rats (94). 16673-34-0, a NLRP3 inflammasome inhibitor derived from glyburide, reduced caspase-1 activity in cardiomyocytes upon LPS (priming) and ATP (activation) stimulation *in vitro*. In a MI mouse model of IRI treatment with 16673-34-0 led to a 40% reduction of IS (95). More recently, OLT1177 (dapansutrile), showed to reduce IS up to 70% in multiple experimental IRI scenarios (short/longer ischemia times, administration during reperfusion and after 1 h of reperfusion) in mice (97). Furthermore, cardiac function 24 h and 7 days after reperfusion was significantly improved compared to placebo and caspase-1 activity in the heart was reduced by 50%. One study tested the effect of NLRP3 inflammasome inhibition in a large animal model of IRI (101). Daily infusion of MCC950, showed to reduce IS up to 16% and preserved cardiac function in a dose-dependent manner in a pig model of 75 min of left anterior descending artery occlusion (101).

In a HTx mouse model, the levels of ASC and IL-1β increased with the progression of cardiac allograft rejection (160). Clinical data is lacking but a small study investigated the hearts of 8 patients with acute transplant rejection and clearly showed formation of ASC (161). Furthermore, a correlation was established between increased ASC formation and rejection grade. Although the study is limited by small numbers these results could indicate that inhibition of the inflammasome has a potential role in the prevention of acute cardiac rejection. Very recently, administration of 16673-34-0 showed a clear improvement in donation after circulatory death (DCD) heart graft function 24 h after implantation in a rat model of HTx (96). One third of the non-treated DCD hearts showed to be necrotic after 24 h, while none of the hearts treated with the NLRP3 inflammasome inhibitor failed.

Results of clinical trials with selective NLRP3 inflammasome inhibitors in myocardial IRI have not been published thus far, although dapansutrile is currently under investigation in multiple phase IB-II clinical trials (98, 99). The compound showed to be safe, was well-tolerated and meaningful plasma levels were obtained in healthy volunteers. Additionally, IL-1β release was clearly reduced in *in vitro* testing on human macrophages (100). Very recently it was studied in a small phase-IIa study of patients with monosodium urate crystal-proven gout flare. Treatment resulted in a reduction of IL-6 and inflammatory pain (162).

Although the results on NLRP3 inflammasome inhibition in small animal IRI models seem very promising, these results should be interpreted with caution. Experimental evidence suggests that divergent effects and potential translation failure could also be the case for the NLRP3 inflammasome inhibitors, since also negative results have been published (163). In NLRP3 KO mice, no protection against IRI was seen in *ex vivo* models or in *in vivo* coronary artery ligation mouse models (164, 165). These findings suggest compensatory mechanisms or, inflammasome independent protective effects of NLRP3 inhibitors (163). Furthermore, deletion of the NLRP3 receptor showed to abolish cardiac ischemic preconditioning in Langendorff-perfused mice hearts (166). This was specific for NLRP3 and not the case for hearts of ASC-deficient mice. In addition, another study, using an *in vivo* mouse model of IRI, reported larger infarcts in mice deficient for NLRP3 and preconditioning did not reduce IS in NLRP3 -or ASC -deficient hearts in contrast to wildtype mice (165).

To justify clinical trials with selective NLRP3 inhibitors in patients with acute MI or HTx it is important to expand our knowledge about the exact mechanisms of the NLRP3 inflammasome and perform translational research in large animal models to fully evaluate the potential of these selective inhibitors.

### P2X Purinoceptor 7

In a healthy cell, ATP is generated intracellularly and is released through transmembrane channels to the extracellular space in a regulated manner. In case of danger, damage and/or stress, however, very high levels of ATP are translocated to the extracellular space, acting as important immunomodulatory DAMPs by activating the P2X purinoceptor 7 (P2X_7_) (167). P2X_7_ activation results in potassium efflux leading to activation of intracellular signaling pathways causing the release of pro-inflammatory cytokines (65). The most investigated function of P2X_7_ in IRI is probably its role in NLRP3 inflammasome assembly and subsequent maturation (65).

In a rat model of MI, it was demonstrated that intramyocardial injections of P2X_7_ specific short hairpin RNA (shRNA) directly after coronary ligation and subsequent MI induction leads to P2X_7_ signaling blockage which resulted in reduced infiltration of circulating cells, a reduction of IS and improved cardiac function. This effect was shown to be associated with inhibiting the Akt and ERK1/2 pathways and NF‐κB activation (67). Another study in rat hearts showed that long non-coding siRNA (NONRATT021972) decreased the upregulation of P2X_7_ in the superior cervical ganglion and improved cardiac function after MI in a rat model of permanent ligation (102). Results that were further supported by a study that investigated (pyridoxalphosphate-6′-azopheny-2′,4′-disulfonate) PPADS, which is a pharmacologic P2X_7_ inhibitor. *In vitro*, PPADS significantly reduced cell death exposed to ischemia. *In vivo*, PPADS reduced IS and improved cardiac function in a mouse model of permanent ligation (68). A rat model of IRI suggests beneficial effects of regulating P2X_7_ as well. Genestin, a flavonoid with anti-inflammatory properties, reduced IS following IRI and was associated with decreased levels of P2X_7_ in myocardial tissue and pro-inflammatory cytokines, such as TNF-α and IL-6, in serum (103).

In HTx, a report showed that P2X_7_ was specifically upregulated in graft-infiltrating lymphocytes in cardiac-transplanted humans and mice. It also showed that short term blocking of P2X_7_ with periodate-oxidized ATP attenuated heart transplant rejection in mice recipients (104). On the contrary, a clinical study highlighted the role of a mutation in P2X_7_ in predicting poor cardiac allograft outcomes. It was demonstrated that cardiac-transplant patients bearing a loss of function mutation for P2X_7_, have a dysregulated P2X_7_/NLRP3 pathway which is associated with higher risk for developing rapidly progressive cardiac allograft vasculopathy and higher frequency of acute rejection episodes (66). This indicates that complete blockage of P2X_7_ may not have favorable outcomes in HTx.

Drugs targeting P2X_7_ were tested in clinical studies for several inflammatory diseases (168–171). Unfortunately, none of these inhibitors have been investigated in the setting of MI or HTx. Novel strategies targeting exATP signaling, particularly through P2X_7_ could have considerable translational potential and novel P2X_7_ inhibitors that are already available for clinical use can also be considered for clinical testing in the setting of MI or HTx.

### Receptor for Advanced Glycation End-Products

In 1992 the Receptor for Advanced Glycation End-Products (RAGE) was first mentioned as a receptor involved in the binding of non-enzymatic glycation products and oxidation of proteins and lipids (14, 172). Further research revealed that RAGE is expressed by multiple cell types including cardiomyocytes, vascular cells, fibroblasts and infiltrating inflammatory cells, and can act as a PRR for a variety of DAMPs, such as HMGB-1 and calcium-binding polypeptides called S100/calgranulins (14). Evidence linking RAGE to MI and IRI dates back to 2006. Knock-out mice for RAGE showed cardioprotective characteristics in the setting of IRI and ischemic rat hearts showed increased RAGE expression. Additionally, pretreatment with pharmacological RAGE blockade, reduced IRI (173).

As mentioned, multiple ligands for RAGE have been identified (e.g. HMGB-1, mtDNA). Additionally, calgranulin also has shown to act on RAGE. Administration of calgranulin in wildtype mice significantly reduced cardiac recovery compared to mice deficient for RAGE, suggesting that activation of RAGE is important for post-MI adverse remodeling and heart failure (69). More recently the treatment of pharmacological blockade with soluble RAGE antagonist (sRAGE/A) and genetic silencing of RAGE was investigated in an *in vivo* rat model of IRI. Treatment with these two strategies synergistically showed a reduction on IS and fibrosis (105). The association between RAGE-mediated signaling following MI in humans has also been reported. AGEs were measured in patients with acute MI and showed to be a predictor for post-MI heart failure, reinfarction and cardiac death (174, 175).

Surprisingly, no increase of plasma AGE or RAGE concentrations was observed in rats in a 4-month follow-up period in the MI group versus a sham group. Furthermore, despite increased RAGE concentrations in the cardiac tissue, no association between early AGE/RAGE levels and cardiac function was observed. These observations indicate that the degree of detrimental effects of RAGE activation differs between animal models and could be attributable to different/unidentified DAMPs activating RAGE directly in the heart (176).

Data on the role of RAGE in HTx is scarce but one study has evaluated its role in a mouse transplant model (177). This study from Moser et al., demonstrated that the intraperitoneal administration of a sRAGE/A, starting one day prior to surgery and daily until sacrifice, had multiple beneficial effects on the cardiac allograft. Graft survival was extended by 2 weeks in the sRAGE/A group compared to the placebo control group in a model of heterotopic, allogeneic HTx. The improved survival was accompanied by attenuated infiltration of inflammatory cells, decreased number of CD3 T cells and reduced apoptosis in the allograft. RAGE-mediated inflammation has also been demonstrated in observational and prospective studies in human following liver transplantation (178, 179).

### Nuclear Factor-Kappa B

Although NF-κB is neither a DAMP nor PRR, it is thought to be a crucial factor in the downstream signaling cascade after PRR activation, especially for TLRs and RAGE. NF-κB dimers are usually located in the cytoplasm as an inactive complex with its natural inhibitor, nuclear factor of kappa light polypeptide gene enhancer in B-cells inhibitor, alpha (IκBα) (180). Activation of NF-κB requires the release of IκBα, followed by a subsequent translocation to the nucleus. The release of IκBα is mediated by IkB kinase (IKK). IKK consists of two catalytic parts; IKK-α and IKK-β, and a regulatory part IKK-γ. After activation by upstream DAMP-PRR ligation, IKK rapidly phosphorylates IkB, which enables the complex to enter the nucleus (180). Upon translocation binding of NF-κB to DNA occurs and gene transcription of inflammatory cytokines is initiated (71, 180).

Studies using rodent models that are deficient for NF-κB or its subunits or mouse models overexpressing IκBα showed a clear reduction of inflammatory cytokine release (IL-1β, IL-6, TNF-α) and smaller IS following myocardial IRI (181–183). Additionally, various (direct and indirect) NF-κB inhibitors have been developed and tested in preclinical models of myocardial IRI (106, 108, 184, 185). BAY 11-7082, a compound that is known to downregulate the activation of NF-κB, was administered in a rat model of IRI and showed to reduce cardiac inflammation and IS (106). Similar effects were observed in mice with BAY 65-1942, an inhibitor that selectively targets IKKβ kinase activity (107). Furthermore, pharmacological inhibition of the proteasome that is needed for the release of IκBα, also significantly preserved cardiac function, reduced IS, and attenuated expression of inflammatory genes in a mouse model of IRI (108). Similarly, administration the calcineurin inhibitor tacrolimus after reperfusion has shown to block the early activation of NF-κB in a rat model of IRI with a subsequent reduction in myocardial necrosis, myeloperoxidase activity, ICAM-1 gene activation and leukocyte accumulation (109).

In HTx, enhanced activity of NF-κB has also been associated with IRI and cardiac allograft rejection although the effects of pharmacological NF-κB inhibition are less studied (186, 187). Nonetheless, pyrrolidine dithiocarbamate (PDTC), a potent inhibitor of NF-κB, did show prolonged longevity of heterotopic cardiac transplants in rats (110). Furthermore, PDTC showed to attenuate IRI after lung transplantation in a pig model (111).

These results suggest a potential role for NF-κB inhibition in myocardial IRI following MI and HTx. In line with many previously discussed therapeutic targets, translation to the clinic so far hast not been successful. A potential reason for hampered clinical translation could be the activation of other transcription factors, parallel to NF-κB after DAMP-PRR interaction. An example of this is interferon (IFN)-regulatory factor 3 (IRF3) translocation after TLR activation. This pathway results in increased cardiac apoptosis and the release of inflammatory cytokines, such as type 1 interferons, which may also play a role in myocardial IRI when NF-κB is inhibited (56, 188, 189). Possible differences regarding the actions of NF-κB after activation of different PRRs (e.g. TLR2, 4, 9 and RAGE) still have to be elucidated.

## Lost in Translation

**Figure 2:**
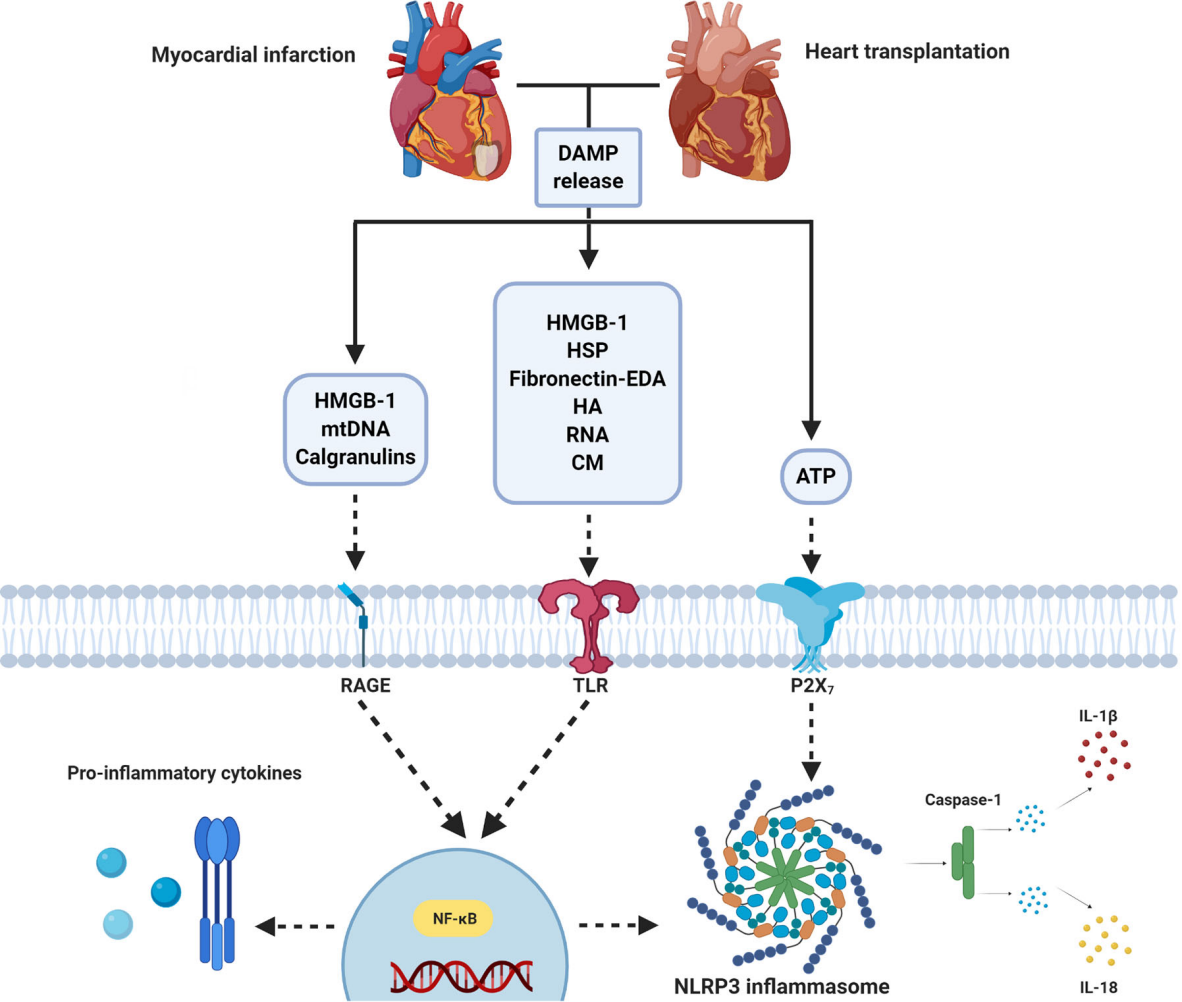
Simplified overview of DAMP/PRR-mediated inflammation following myocardial infarction and heart transplantation. Ischemia/reperfusion following MI and heart transplantation leads to the release of DAMPs (e.g. ATP, HMGB-1, HSP, mtDNA), which act on PRRs (e.g. TLRs, RAGE and P2X_7_). Stimulation leads to nuclear migration of NF-κB. This results in the production of pro-inflammatory cytokines and NLRP3 inflammasome activation. ATP, Adenosine Triphosphate; CM, Cardiac myosin; DAMP, Damage associated molecular pattern; EDA, Extra Domain A; HA, Hyaluronic acid; HMGB-1, High mobility group box-1; HSP, Heat shock protein; IL, interleukin; NLRP3, NOD- Leucine-Rich Repeat- and pyrin domain-containing protein 3; mtDNA, Mitochondrial DNA; NF-κB, Nuclear Factor kappa-light-chain-enhancer of activated B cells; P2X_7_, P2X purinoceptor 7, RAGE, Receptor for Advanced Glycation End products; RNA, Ribonucleic Acid; TLR, Toll-like receptor. Figure was created with BioRender.com.

**Figure 3:**
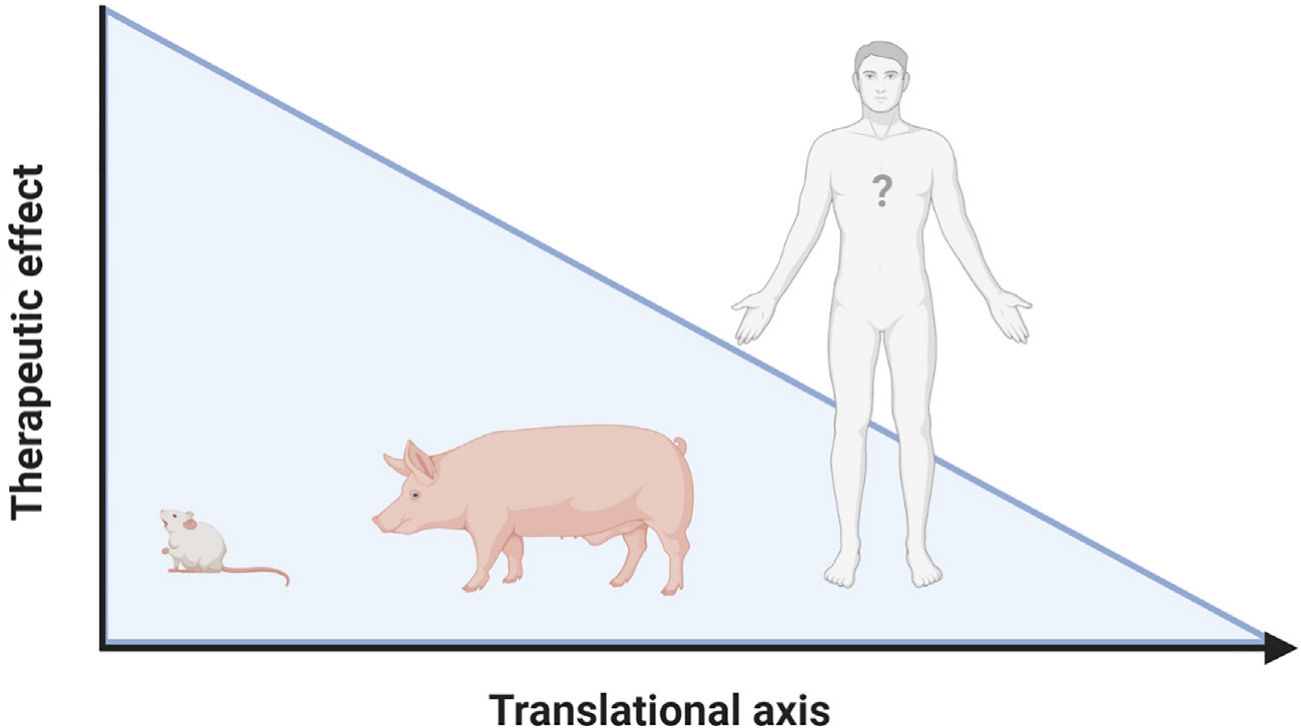
Lost in translation. Reduced effectiveness of therapies targeting the inflammatory response following IRI when moving along the translational axis is a constant in literature. Possible explanations for this translational failure are the incredibly heterogeneous models used, the complexity of the cardiac patient, the lack of knowledge on DAMP/PRR mediated signaling in humans and the “Janus face” that many of the mediators have. Figure was created with BioRender.com.

The inflammatory response following IRI upon MI and HTx is orchestrated by a complex interplay between DAMPs and PRRs that are released from, and bound to multiple cardiac and circulating cell types (**Figure 2**). Years and years of research showed the cardioprotective potential of targeting these mechanisms in experimental small animal models and tried to unravel the immense complexity of the mechanisms at hand. To date, however, none of these promising treatments have been implemented in standard clinical treatment strategies (9, 74). A reduction in the effectiveness of cardioprotective therapies when moving along the translational axis, from small to large animal models, is well described in the literature (190–192). Multiple explanations of this so-called translational failure can be provided (**Figure 3**).

Animal models used in the setting of IRI in MI are numerous and incredibly heterogeneous (193). Mice are most used for obvious reasons, but great variety exist even within this species regarding coronary anatomy, IS as a percentage of the area at risk and overall inflammatory response initiated by damage to the myocardium (194). Furthermore, many different techniques of coronary occlusion are available and some of these techniques (open vs. closed chest) directly influence systemic inflammation and thus could affect treatment results (194, 195). The duration of myocardial ischemia also greatly differs and is known to influence treatment efficacy. Additionally gender, age and housing conditions of mice and other species are known to influence anti-inflammatory treatment efficacy in the setting of MI (196). In line with this, the animal models used to study HTx are often far from clinical reality. In rodents, most studies are performed with heterotopic transplantation, in which the donor heart is connected to the native heart in a parallel fashion. This approach is followed to increase the survival of the recipient and maintain circulation in case of severe acute rejection. Nowadays, this technique is rarely used in the clinical setting since orthotopic transplantation is preferred. A better strategy to study the involvement of DAMPs and PRRs in IRI following HTx may be found in using ex vivo heart machine perfusion (EVHP). Using healthy, physiologically relevant, pig hearts or rejected transplant hearts in the EVHP system allows to better reflect the human situation and to study inflammatory mechanisms and effect of therapeutics in more detail (197, 198). Additionally, ischemia time is an important factor in IRI and transplantation outcome (199). In the clinical setting, the average ischemia time for HTx is 4 h and never exceeds 6 h whereas in rodent models the studied ischemia time varies from 0 to 8 h. Moreover, there is no clear consensus on administration route, treatment frequency or duration. Taken together, these factors make studies difficult to compare and hampers translation of positive findings directly to clinical therapies.

Accordingly, given the heterogeneity of current animal models used to study cardiac IRI, it is reasonable to assume that the inflammatory response following MI and HTx differs between animal species. It is not unlikely that dominant PRRs and their function differ between mice, pigs and humans. It is therefore important and essential to further expand our knowledge on the release of DAMPs and PRR expression in large animals and humans. There are major differences between the preclinical models used and the actual patient. In patients with severe atherosclerosis and comorbidities, such as diabetes and obesity, a chronic low grade level of inflammation with concomitant activation of the innate immune response, either local or systemic, is already present (200). For example, the NLRP3 inflammasome is activated in stable atherosclerotic disease by cholesterol crystals and oxidized low-density lipoprotein (201). It is not clear whether this chronic systemic and local “activation” could result in reduced or increased impact of the NLRP3 inflammasome and/or other DAMP-PRR interaction. The inflammatory response in atherosclerotic patients, however, is most likely different than in healthy animals with non-atherosclerotic arteries. Additionally, we cannot rule out the possibility that other therapies, that are already used as a standard cardiac treatment (aspirin, beta-blockers, ACE-inhibitors, statins) or during HTx (immunosuppressors as Tacrolimus, Sirolimus and Cyclosporin-A), diminish beneficial effects of inhibiting PRRs, DAMPs and immune responses. Statins, for example, exert inhibitory effects on inflammasome and TLR activation (202). Future research should therefore also focus on the role and effects of co-medication on cardioprotective pathways and the interferences with potential new drugs. Ideally, in animal models, experimental therapies should be tested on top of optimal medical treatment regimes, if possible, in an atherosclerotic background.

A possible explanation for translational failure is the “Janus face” that many of these DAMPs and PRRs have. DAMPs like HMGB-1 and HSPs are involved in both protective and harmful signaling following IRI and MI. Similar observations were made for PRRs, such as TLRs and NLRP3. The inflammatory response after myocardial IRI can both be thought of as a functional, since cell debris needs to be removed and as detrimental, since viable myocardium can potentially be permanently damaged. The inflammatory response should therefore be carefully ‘fine-tuned’ rather than rigorously altered (203). The influence of certain DAMPs in IRI is also time-dependent. This is clearly illustrated by the role of HMGB-1, where different effects of inhibition in the pre- and post-ischemic phase where observed (113). Furthermore, the activity of the NLRP3 inflammasome seems to increase between 1 and 3 h post reperfusion and IS was not increased by NLRP3 inflammasome mediated signaling within the first hour after ischemia (156). This implicates that both timing and treatment duration are of vital importance for study outcome and could be explanatory for contradictory results. Importantly knock-out and pretreatment studies may be pivotal for mechanistic insights but are not compatible with clinical treatment protocols. Pretreatment with an agonist (preconditioning) to a certain receptor could lead to similar effects as posttreatment with an antagonist and in part explains the observed contradictory results in this line of research. Results from preclinical models that are incompatible with clinical disease course should therefore be interpreted with great caution.

Effective treatments have the aim to either prevent DAMP release or target the released DAMPs (200). When choosing a therapeutic strategy it is important to realize that different forms and locations of certain DAMPs can have contradicting effects. For example, extracellular HSP60 showed clear detrimental effects during IRI, but just recently intracellular HSP60 showed to be essential for normal mitochondrial and cardiomyocyte homeostasis, indicating that complete blockage or deletion of HSP60 is not a feasible cardioprotective strategy (204). In addition, the ability of HMGB-1 to bind certain PRR’s and the subsequent inflammatory response following IRI, highly relates to the ability of the different redox states of HMGB-1 (113). Hence, this indicates that HMGB-1 inhibition should be selective to the redox forms that are only involved in damaging inflammatory signaling. In order to be able to specifically target DAMPs in the context of IRI, the impact of different active forms and their specific cellular location, at different time points following reperfusion should be better established.

Since PRRs are activated by multiple DAMPs, one could hypothesize that it is more attractive to target PRRs directly rather than targeting specific DAMPs. The different PRRs, however, share common ligands for activation and are multifunctional. It is insufficiently investigated if the released DAMPs that cannot bind the single inhibited PRR, as a consequence, activate and mediate downstream inflammatory pathways of other PRRs. Further research should elucidate what the effect is of specific PRR inhibition on other DAMP-PRR interactions. Given the clear level of interplay between the different inflammatory pathways and the complexity of IRI in patients it has been hypothesized that, to effectively target IRI, we should try multitargeted approaches. A combination of an anti-inflammatory drug with a drug targeting other pathways that lead to cardiomyocyte death, or one that activates endogenous cardioprotective pathways was proposed (205). Given the high level of cross-talk between DAMPs and PRR’s we could, also think of combined approaches that target both PRRs and DAMPs. This combined DAMP/PRR approach should be evaluated in animal models before heading to the clinic, considering the higher chance that crucial pathways for recovery could be blocked as well.

## Conclusion and Future Recommendations

In conclusion, extensive research led to the identification of multiple molecules that act as DAMPs on several PRRs. This upstream interaction is thought to be crucial for a pro-inflammatory milieu that significantly contributes to the harmful effects of IRI in MI and HTx. This signaling cascade has been subject to development of novel therapeutic approaches for decades now. Nevertheless, the road to clinical translation has not been straightforward.

Future studies targeting DAMP/PRR mediated inflammation should therefore be performed in multiple, standardized, clinically relevant animals before proceeding to the clinical arena. Additionally, it is essential to ensure reproducibility at different locations and in multiple scenarios. Translational research should focus on further unraveling the complex interaction and level of cross-talk that many of these mediators have. Finally, human studies that investigate if these mechanistic pathways play similar roles in myocardial IRI are urgently warranted to underline that these studied mechanisms in animal models are also essential in the cardiac patients we aim to treat.

## Author Contributions

MS reviewed the literature and wrote the first version of the manuscript. SK provided the background of the HTx field and together with CO and MM helped with a proper literature search and the writing of the manuscript. NK, PD, JS, DK, SJ, LB, and GH reviewed the manuscript intensively. GH edited the manuscript and conceived the original idea of the project. All authors contributed to the article and approved the submitted version.

## Funding

This work was supported by the European Union’s Horizon 2020 research and innovation program under the Marie Sklodowska-Curie grant agreement (RESCUE, No 801540), is supported by Horizon2020 ERC-2016-COG EVICARE (725229), and by the partners of Regenerative Medicine Crossing Borders (www.regmedxb.com) and powered by Health∼Holland, Top Sector Life Sciences & Health.

## Conflict of Interest

The authors declare that the research was conducted in the absence of any commercial or financial relationships that could be construed as a potential conflict of interest.

## Abbreviations

AGEs, advanced glycation end products; ASC, apoptosis-associated speck like protein containing a caspase-recruitment domain; ATP, adenosine triphosphate; cfDNA, cell-free deoxyribonucleic acid; CM, cardiac myosin; CpG, deoxycytidylate-phosphate-deoxyguanylate; DAMPs, damage-associated molecular patterns; Dex-TO, dextran carrier conjugated to thiazole orange; DNA, deoxyribonucleic acid; EDA, extra domain A; EGCG, epigallocatechin-3-gallate; EVHP, *ex vivo* heart perfusion; exATP, extracellular ATP; exRNA, extracellular RNA; GGA, geranylgeranylacetone; GL, gycyrrhizin; HA, hyaluronic acid; HMGB-1, high mobility group box-1; HSPs, heat shock proteins; HTx, heart transplantation; IkBα, nuclear factor of kappa light polypeptide gene enhancer in B-cells inhibitor, alpha; IKK, IkB kinase; IL, interleukin; IRI, ischemia reperfusion injury; IS, infarct size; LPS, lipopolysaccharides; MI, myocardial infarction; mtDNA, mitochondrial DNA; NF-κB, nuclear factor kappa-light-chain-enhancer of activated b cells; NLRP3, NOD-, LRR- and pyrin domain-containing protein 3; NLRs, NOD-like receptors; NOD, nucleotide-binding oligomerization domain; PPADS, pyridoxal phosphate-6’-azopheny-2’,4’-disulphonate; PRRs, pattern recognition receptors; P2X_7_, P2X purinoceptor 7; RAGE, receptor for advanced glycation end products; RNA, ribonucleic acid; ROS, reactive oxygen species; shRNA, short hairpin RNA; siRNA, small interfering RNA; sRAGE, soluble RAGE antagonist; STEMI, ST-elevated myocardial infarction; TAK-242-NP incorporation of TAK-242 into poly-(lactic-co-glycolic acid) nanoparticles; Th17, T helper cells type 17; TLRs, Toll-like receptors; TNF-α, tumor necrosis factor-α.
